# Current routines for antibiotic prophylaxis prior to transrectal prostate biopsy: a national survey to all urology clinics in Sweden

**DOI:** 10.12688/f1000research.19260.1

**Published:** 2020-01-28

**Authors:** Johan Styrke, Sven Resare, Karl-Johan Lundström, Patrick Masaba, Christofer Lagerros, Pär Stattin

**Affiliations:** 1 Department of Surgical and Perioperative Sciences, Urology and Andrology, Umeå University, Umeå, 901 87, Sweden; 2 Department of Urology, Sundsvall Hospital, Sundsvall, 851 86, Sweden; 3 Department of Surgical Sciences, Uppsala University, Uppsala, 752 36, Sweden

**Keywords:** Prostate biopsy, Antibiotic prophylaxis, Prostate cancer diagnostics, Survey

## Abstract

**Background: **The risk of infection after transrectal ultrasound (TRUS)-guided prostate biopsies is increasing. The aim of the study was to assess the use of antibiotic prophylaxis for prostate biopsy in Sweden.

**Methods: **All public and private urology clinics reporting to the National Prostate Cancer Register of Sweden received a survey on TRUS-biopsy prophylaxis.

**Results: **Of the 84 clinics surveyed, 76 replied (90%). If no risk factors for infection were present, a single dose of ciprofloxacin 750 mg was used by 50 clinics (66%). Multiple doses of ciprofloxacin 500 or 750 mg (n=14; 18%) or a single dose of trimethoprim-sulfamethoxazole 160/800 mg (n=7; 9%) were other common prophylaxes. Most clinics gave the prophylaxes immediately before the biopsy (n=41; 54%). Urine dipstick was used by 30 clinics (39%) and rectal enema by six (8%). In patients with high risk of infection, the survey mirrors a large variety of regiments used.

**Conclusions: **The preference to use a single dose of ciprofloxacin 750 mg is in accordance with the Swedish national guidelines for patients with a low risk of infection. Better compliance to the guideline recommendation to use a urine dipstick would probably increase the number of patients classified as having an increased risk of infection. Being classified as a high-risk patient should lead to an extended duration of antibiotic prophylaxis, however, the variety of regimens used in the high-risk group reflects an inability to treat these patients in a standardized fashion and also highlights a need for more clear-cut guidelines. Pre-biopsy identification of high-risk patients is an important issue to tackle for the urologic clinics in order to reduce the number of infections.

## Introduction

Common side effects following transrectal ultrasound-guided prostate biopsy (TRUS-biopsy) include urinary tract infections (UTI) sometimes leading to hospitalization ^1^. In Sweden, about 6% of the patients have a prescription of antibiotics during the first month following TRUS-biopsy and about 1% are hospitalized ^2^. The infection rate can be reduced by the use of prophylactic antibiotics, or bowel cleansing with povidone-iodine ^3– 7^. The use of antibiotic prophylaxis is underscored by the European, American and new national Swedish guidelines ^8– 10^. The most commonly used antibiotics are fluoroquinolones in various regimens ^3, 11, 12^.

A rising problem is the presence of drug-resistant bacteria leading to increased post-biopsy infection rates ^13^, especially fluoroquinolone-resistant *Escherichia coli* is problematic and have been assessed to explain over 40% of TRUS-biopsy infections in the USA ^14^. In Europe, fluoroquinolone resistance is found in 8–46% of *E. coli* isolates, the former figure is the Swedish rate ^15, 16^. Resistance to trimethoprim-sulfamethoxazole is found in about 20% of isolates in Sweden ^16^. The rising resistance calls for studies aiming at identifying new suitable antibiotics or new ways of reducing the use of fluoroquinolones. There is also a need for strict adherence to guidelines to avoid overuse of antibiotics and to better identify risk groups for infection, i.e. patients with indwelling catheter, patients with a urine dip-stick positive for nitrite, patients with previous urinary tract infection, diabetes or immunosuppressive treatment according to the national Swedish guidelines, first published in April 2014 ^10^. Patients with risk factors for infection show slightly higher infection rates than those without ^2^. A recent study concluded that adherence to the European Association of Urology (EAU) guidelines on prophylactic antibiotics safely can reduce the use of antibiotics and lower resistance rates ^17^.

The primary aim of the present study was to describe the type and timing of antibiotics used prior to TRUS-biopsy in Sweden in low- and high-risk patients and to investigate to what extent urine dipstick, urine culture and rectal enema is used. The secondary aims were to investigate if the antibiotic strategy has changed during 2006–2014 and to compare adherence to the Swedish national guidelines between university hospital departments, non-university hospital departments and private practitioners.

## Methods

### Survey

An electronic survey (available as *Extended data* ^18^) was distributed to all of the hospitals and outpatient urology clinics reporting to the national Swedish National Prostate Cancer Register (NPCR). The register captures 98% of all prostate cancer cases when compared to the mandatory national cancer register ^19^. The web-based Information Network for CAncer registers in Sweden (INCA) platform was used for reporting. Recipients of the survey were the trained staff reporting to the NPCR or the heads of department if contact with the staff could not be established. In one case, where neither of these recipients could be reached, the survey was distributed to a urologist known by the authors at the clinic in question. The questionnaire comprised six questions concerning current standard prophylaxis in patients with and without risk factors for infection, time of administration, the use of urine dipstick, urine culture or rectal enema prior to TRUS-biopsy during 2006–2014 ( Table 1– Table 3). All of the questions were followed by a question regarding if, and when, strategies had been altered during 2006–2014 ( Table 4). The questionnaires were distributed 2014-11-26 and after up to three reminders per e-mail, the last response was collected 2015-11-03.

**Table 1.** Drug of choice and time of administration in patients without risk factors for infection. The table displays the answers from urology departments in Sweden in late 2014 / early 2015 regarding their current use of antibiotic prophylaxes prior to prostate biopsy.

Question 1a: What prophylaxis is currently used as standard at your department?		
	n	%
**a.** Ciprofloxacin 750 mg x 1	50	66
**b.** Ciprofloxacin 500 mg x 1	2	3
**c.** Multiple doses of ciprofloxacin (500 or 750 mg)	14	18
**d.** Trimethoprim-sulfamethoxazole 160/800 mg x 1	7	9
**e.** Multiple doses of trimethoprim-sulfamethoxazole	1	1
**f.** Other, please specify	2	3
Question 2a: At what point do you give the antibiotics to the patient?		
	n	%
**a.** Immediately before the biopsy	41	54
**b.** Immediately after the biopsy	12	16
**c.** More than 1 h prior to the biopsy	8	11
**d.** Before and after the biopsy	12	16
**e.** Other, please specify	1	1
Non-responders	2	3

**Table 2.** Use of urine dipstick, urine culture as routine and rectal enema. The table displays the answers from urology departments in Sweden in late 2014 / early 2015 regarding their current routines in identifying high-risk patients with urinary tract infections prior to prostate biopsy and also if rectal enema is used.

Question 3a: Is urine dipstick currently used at your department prior to TRUS-biopsy?		
	n	%
**a.** Yes	30	39
**b.** No	41	54
Non-responders	5	7
Question 4a: Is urine culture currently used at your department prior to TRUS-biopsy?		
	n	%
**a.** Yes	3	4
**b.** No	69	91
Non-responders	4	5
Question 5a: Is rectal enema currently distributed prior to TRUS-biopsy at your department?		
	n	%
**a.** Yes	6	8
**b.** No	67	88
Non-responders	3	4

**Table 3.** Duration of treatment for patients with elevated risk of infection. The table displays the answers from urology departments in Sweden in late 2014/early 2015 regarding their current use of antibiotic prophylaxes prior to prostate biopsy in high-risk patients.

Question 6a: Specify how antibiotic prophylaxis is used in patients with risk factors of infection (patients with indwelling catheter, a urine dip-stick positive for nitrite, previous urinary tract infection, diabetes or immunosuppressive treatment)		
	n	%
**a.** Same strategy as for the low-risk patients	11	15
**b.** Prolonged prophylaxis (>1 dose, <4 days) without a urine culture	19	25
**c.** Prolonged prophylaxis (>1 dose, <4 days) according to a urine culture	1	1
**d.** Treatment ≥4 days without a urine culture	13	17
**e.** Treatment ≥4days according to a urine culture	9	12
**f.** Alternative b or c	10	13
**g.** Alternative d or e	6	8
**h.** Other, please specify	2	3
Non-responders	5	7

**Table 4.** Changes in routines for patients with elevated risk of infection. The table displays the answers from urology departments in Sweden in late 2014 / early 2015 concerning their changes in routines prior to prostate biopsy during 2006-2014 with respect to drug of choice, time of administration, use of urine dipstick, use of urine culture, use of rectal enema and duration of treatment.

Question 1b: Have you changed the drug of choice during 2006–2014?		
	n	%
**a.** Yes ^a^	22	29
**b.** No	42	55
**c.** Unable to recall	8	11
Non-responders	4	5
Question 2b: Have you changed the time of administration during 2006–2014		
	n	%
**a.** Yes ^b^	16	21
**b.** No	48	63
**c.** Unable to recall	8	11
Non-responders	4	5
Question 3b: Have you changed the use of urine dipstick during 2006–2014?		
	n	%
**a.** Yes ^c^	12	16
**b.** No	57	75
**c.** Unable to recall	2	3
Non-responders	5	7
Question 4b: Have you changed the use of urine culture during 2006–2014?		
	n	%
**a.** Yes ^d^	3	4
**b.** No	67	88
**c.** Unable to recall	1	1
Non-responders	5	7
Question 5b: Have you changed the use of rectal enema during 2006–2014?		
	n	%
**a.** Yes ^e^	1	1
**b.** No	70	92
**c.** Unable to recall	3	3
Non-responders	3	4
Question 6b: Have you changed the duration of treatment in patients with elevated risk of infection during 2006–2014?		
	n	%
**a.** Yes ^f^	7	9
**b.** No	57	75
**c.** Unable to recall	8	11
Non-responders	4	5

^**a**^Ten had reduced the amount of prophylaxis from multiple- to single dose regimens, one had changed the dose of a single ciprofloxacin administration, six had changed from trimethoprim-sulfamethoxazole to ciprofloxacin, three had changed from ciprofloxacin to trimethoprim-sulfamethoxazole, one had changed from amoxicillin to ciprofloxacin and one had changed regimen but could not recall how. ^**b**^Thirteen had changed from early administration to administration immediately prior to the biopsy and two had done the opposite change, one had changed but could not recall how. ^**c**^Ten had introduced urine dipstick as routine on all patients, one had stopped using urine dipstick and instead used urine culture on all patients, one had changed but could not recall how. ^**d**^Three had introduced routine urine culture during the study period. ^**e**^One had quit using enema, two had decided to introduced enema in 2015. ^**f**^Three had adopted to the guidelines in 2014, one had introduced a checklist in 2012, two had adopted a “more active strategy” and one had changed strategy but could not recall how.

### Statistical calculations

Data from the survey was downloaded into Microsoft Excel 2011 (Microsoft Corp., Redmond, WA) and exported to SPSS Statistics 23 (SPSS Inc., Chicago, IL) for further analysis. Standard descriptive statistics were used to present the results. A comparison was conducted between university hospital departments, non-university hospital departments and private practitioners for adherence to the national Swedish guidelines ^10^ defined as using a single dose of prophylaxis to the low-risk group, using multiple dose regimens to the high-risk group and analysing urine dipstick for nitrite prior to biopsy. The Likelihood Ratio-test (G2) was used. Statistical significance was defined as p<0.05. All missing data are presented in Table 1– Table 4.

### Ethics

Patient data were not investigated in the present study. An ethics approval was still obtained from the local ethics committee in Umeå, no 2016-228/31. The committee approved the research project according to the application.

## Results

### Survey response

**Figure 1.**
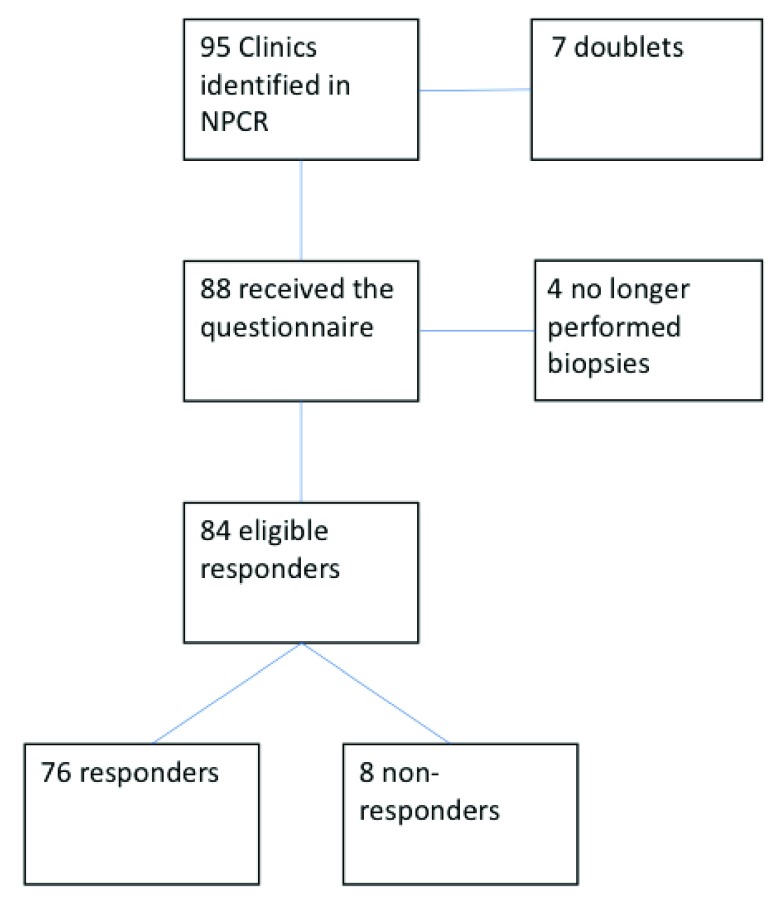
Flow chart of the selection of clinics. NPCR, The Swedish National Prostate Cancer Register.

The survey was sent to 84 recipients and answers were obtained from 76 of the clinics (90%) ( Figure 1). Of these, seven were university hospital departments, 47 were non-university public hospital departments, two were hospital departments owned by a private company, one was a hospital department owned by a foundation and 19 were private practitioners. De-identified survey responses are available as *Underlying data* ^18^.

### Use of prophylaxis

In patients without risk factors for infection the most frequently used antibiotic was a single dose of ciprofloxacin 750 mg used by 50 clinics (66%). The second and third most common choices were multiple doses of ciprofloxacin 500 mg or 750 mg used by 14 clinics (18%) and a single dose of trimethoprim-sulfamethoxazole 160/800 mg used by seven clinics (9%). The prophylaxes were distributed immediately before or immediately after the biopsy in 41 (54%) and 12 (16%) of the clinics respectively, more than one hour before the biopsy in 8 (11%) and both before and after the biopsy in 12 clinics (16%) ( Table 1). A single dose of ciprofloxacin 750 mg distributed as recommended by the national guidelines—immediately before or more than one hour before the biopsy—was used by 39 clinics (46%).

Urine dipstick was used by 30 clinics (39%), urine culture as routine for low-risk patients by three clinics (4%) and rectal enema by six clinics (8%) ( Table 2). In the question regarding the high-risk group, the results were mixed; 19 clinics used prolonged prophylaxis (>1 dose, <4 days), 13 clinics (17%) used treatment ≥4 days without a urine culture and 11 clinics (15%) utilized the same strategy as for the low-risk patients (of which seven only used single dose prophylaxis) ( Table 3).

There were 10 clinics (13%) that reduced the amount of antibiotic prophylaxis from multiple- to single-dose regimens during the study period; six (8%) had changed from trimethoprim-sulfamethoxazole to ciprofloxacin and three (4%) had done the opposite. A further 13 (17%) had changed from early administration to administration immediately prior to the biopsy and 10 (13%) had introduced urine dipstick as routine on all patients ( Table 4).

**Figure 2.**
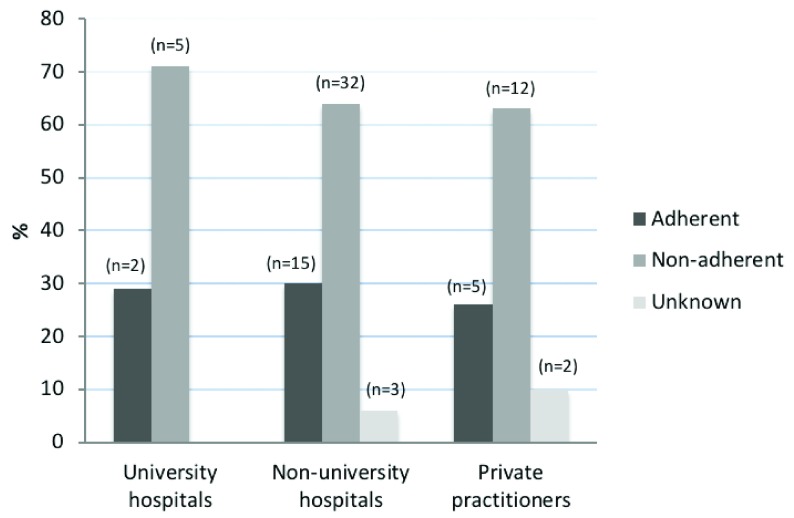
Adherence to guidelines in university hospital departments, non-university hospital departments and private practitioners. The figure displays summarized answers from urology departments in Sweden in late 2014/early 2015 regarding their current adherence to guidelines for antibiotic prophylaxes prior to prostate biopsy. Adherence to guidelines is defined as using a single dose of prophylaxis to the low-risk group, using multiple dose regimens to the high-risk group and analysing urine dipstick for nitrite prior to biopsy.

When comparing university hospital departments, non-university hospital departments and private practitioners for adherence to guidelines, no significant differences were found ( *p*=0.8) ( Figure 2).

## Discussion

In the present study, the self-reported antibiotic prophylaxis standard patterns for transrectal prostate biopsies in Sweden 2006–2014 are reported. The web-based survey covered 90% of clinics diagnosing prostate cancer in Sweden.

The first published study to investigate the clinical routines for TRUS-biopsies in Sweden in 2011 addressed the regular procedure in a standard case scenario and did not account for patients with elevated infection risk ^20^. The preferred antibiotic prophylaxis consisted of ciprofloxacin at the time of biopsy (64%) in patients without risk factors for infection, which is comparable to our results (70%). However, dosage and exact timing of administration (i.e. immediately before or after biopsy) were not inquired. Trimethoprim-sulfamethoxazole was the second most common alternative in the respective studies (12% vs 9%). The similarities between the studies are not surprising given the overlapping time periods. The use of fluoroquinolones as standard is supported by two meta-analyses showing that fluoroquinolones have significantly better effects compared with placebo in all included trials ^3, 7^. Other antibiotic agents that have been investigated alone or in addition to fluoroquinolones include trimethoprim-sulfamethoxazole, gentamicin, fosfomycin, piperacillin-tazobactam, amikacin, ceftriaxone, amoxicillin-clavulanate and meropenem ^3, 6, 7, 20– 24^. Most regiments appear to reduce the risk of infection but there is a lack of sufficiently powered randomized trials comparing fluoroquinolones to other antibiotic classes ^7^. The most recent meta-analysis, however, concludes that the use of augmented antibiotics might be beneficial ^25^. None of these alternatives have been evaluated in a Swedish setting with relatively low resistance. Diversification of substances for antibiotic prophylaxis depend on information about efficacy obtained from randomized trials complemented with local bacterial resistance patterns and possibly information obtained by the use of rectal swabs—a strategy supported by a number of studies ^26, 27^.

According to the present study, most urology departments give the antibiotic prophylaxis immediately before the prostate biopsy. This strategy is supported by a study by Lindstedt *et al*. prospectively comparing 1322 biopsies in 1161 patients from two nearby hospitals. At one of the hospitals, 750 mg ciprofloxacin was given two hours prior to the biopsy and at the other hospital the same prophylaxis was given immediately before the biopsy. The results revealed no significant differences between the groups in both of which hospital admission for febrile urinary tract infection (UTI) occurred in less than one per cent of the cases ^28^. Owing to the low number of febrile UTI (n=12 in total) the possibility that the study was underpowered to find a small significant difference cannot be ruled out; the study was also not randomized, making the level of evidence lower. These results have been extrapolated to determine the timing also of trimetoprim-sulfamethoxazole prior to TRUS-biopsy in Sweden. Future randomized trials are needed to investigate this issue further. Two meta analyses have not shown significant differences in risk of infection when comparing single versus multiple doses of antibiotics ^3, 7^.

A urine dipstick test is useful for bacteriuria screening if the results of both nitrites and leukocyte-esterase are negative ^29^; however, a small number of false-negative tests will occur ^28^. Prior urine bacterial culture does not seem to be advantageous in patients without risk factors for infection ^28, 30^. However, a more stringent implementation of a dipstick urine sample may allow identifying more patients at risk for infection. As a consequence, patients would have to undergo urine culturing prior to TRUS-biopsy which may also possibly reduce the risk for prophylactic antibiotic failure.

In patients with risk factors for infectious complications the clinical practice varied in our study with the most widespread strategy being an extended duration of antibiotic prophylaxis (42%). This is somewhat in line with the EAU guidelines on urological infection favouring an individual approach in patients at risk ^8^. The variety of prophylactic regimens also reflects the difficulties in identifying these patients in a standardised fashion. Risk factors for infection outlined in the national Swedish guidelines include a positive urine dip stick or urine culture, previous febrile infections following prostate biopsy, previous urinary tract infections or bacterial prostatitis, diabetes, immunosuppression or an indwelling bladder catheter ^10^. Other risk factors that have been proposed are presence of fluoroquinolone resistant *E. coli*, old age, previous prostate biopsy, hospitalization prior to biopsy and non-adherence to antibiotic prophylaxis ^6, 13^. The rising bacterial resistance to fluoroquinolones in the rectal flora over time has led to a rise in infectious complications ^1, 6, 27^. Patients who have previously been treated with fluoroquinolones have been identified as a group at risk for harbouring bacteria with fluoroquinolone resistance ^31^. The current national Swedish guideline does not address this risk group explicitly. A systematic and thoroughly taken patient history accounting for the presence of these risk factors must be emphasized as a measure of importance to find patients at high risk of infection. A better adherence to the guidelines for low-risk patients may similarly reclassify patients to the high-risk group. There is a need for studies aiming at reducing the risk of infection as well as reducing the amount of antibiotics used in the high-risk group.

That only a minority of the clinics adhere to the national Swedish guidelines might be explained by the fact that the guidelines were first introduced in April 2014. Hopefully the figures will improve over the years to come.

The main weakness of the study is that the answers to the questionnaire reflect the official policy of each clinic and are not provided by individual doctors, as strategies may differ between doctors within the same clinic. It is, however, likely that there are local routines used by most urologists—at least regarding the low-risk group as there are well established guidelines for this group ^10^. The antibiotic strategy in high-risk patients probably varies more and the lack of clear-cut national guidelines may lead to a more individual approach by urologists. Regarding the questions about regimens changes during 2006–2014, there is a risk of recall bias. The survey was constructed by the authors before being sent out and is not validated. It was conducted in late 2014; it is, however, likely that most clinics still use the strategies shown in the survey because no major changes in the national or European guidelines for prostate cancer have emerged since then. The strength of the study is that it includes 90% of all clinics conducting prostate biopsies in Sweden. The results only apply to Swedish conditions but due to the high response rate the internal validity is assessed to be high.

In conclusion, the preference to use a single dose of ciprofloxacin 750 mg is in accordance with the Swedish national guidelines for patients with a low risk of infection. Better compliance to the guideline recommendation to use a urine dipstick would probably increase the number of patients classified as having an increased risk of infection. Being classified as a high-risk patient should lead to an extended duration of antibiotic prophylaxis, however, the variety of regimens used in the high-risk group reflects an inability to treat these patients in a standardized fashion and also highlights a need for more clear-cut guidelines. Pre-biopsy identification of high-risk patients is an important issue to tackle for the urologic clinics in order to reduce the number of infections.

## Data availability

### Underlying data

Swedish National Data Service: Current routines for antibiotic prophylaxis prior to transrectal prostate biopsy – a national survey to all urology clinics in Sweden. https://doi.org/10.5878/zdne-z984 ^18^.

SND1137-001-V1.0.zip contains the following underlying data:

- Data_Survey_TRUS-biopsy-prophylaxis_Sweden.csv (results of the survey in CSV format).
- Data_Survey_TRUS-biopsy-prophylaxis_Sweden.xlsx (results of the survey in Microsoft Excel format).
- Variable_list_TRUS-biopsy-prophylaxis_Sweden.pdf (list of variables used in the dataset).

### Extended data

Swedish National Data Service: Current routines for antibiotic prophylaxis prior to transrectal prostate biopsy – a national survey to all urology clinics in Sweden. https://doi.org/10.5878/zdne-z984 ^18^.

SND1137-001-V1.0.zip contains the following underlying data:

- Survey_TRUS-biopsy-prophylaxis_Sweden.pdf (copy of the survey in English).

Data are available under the terms of the Creative Commons Zero "No rights reserved" data waiver (CC0 1.0 Public domain dedication).
