# Supplementary material for: Current routines for antibiotic prophylaxis prior to transrectal prostate biopsy: a national survey to all urology clinics in Sweden
Source: F1000Res. 2020 Jan 28;9:58. [Version 1] doi: 10.12688/f1000research.19260.1 (PMC7194489; doi:10.12688/f1000research.19260.1)
Supplement: Peer review history

# Peer review: Reviewer response for version 1

**DOI:** 10.5256/f1000research.21108.r62141

**Published:** 2020-04-20

**Authors:** Shingo Yamamoto

**Affiliations:** 1 Department of Urology, Hyogo College of Medicine, Nishinomiya, Japan

This report is important to know the real world data in terms of prophylaxis for TRUS in Sweden. However, several points are to be discussed as below.

1. Infection following TRUS is not only UTI but mainly prostatitis. 
2. Thus, by previous papers and guidelines, risk factors are defined as antimicrobial-resistant bacterial flora in the rectum as as well as bacteriuria  in the bladder.
3. Recently several papers reported that the culture of rectal swab is useful to prescribe prophylaxis for TRUS.
4. Some papers recommend parenteral antimicrobials, especially in high risk patients.

Is the work clearly and accurately presented and does it cite the current literature?

Yes

If applicable, is the statistical analysis and its interpretation appropriate?

Yes

Are all the source data underlying the results available to ensure full reproducibility?

Yes

Is the study design appropriate and is the work technically sound?

Yes

Are the conclusions drawn adequately supported by the results?

Partly

Are sufficient details of methods and analysis provided to allow replication by others?

Yes

Reviewer Expertise:

urinary tract infections

I confirm that I have read this submission and believe that I have an appropriate level of expertise to confirm that it is of an acceptable scientific standard.

# Peer review: Reviewer response for version 1

**DOI:** 10.5256/f1000research.21108.r59268

**Published:** 2020-02-14

**Authors:** Truls Bjerklund-Johansen

**Affiliations:** 1 Department of Urology, Oslo University Hospital, Oslo, Norway

The paper reports that fluoroquinolone based prophylaxis is most commonly used in transrectal prostate biopsies in Sweden and that extended prophylaxis is often used if the patient has risk factors for infective complications.

 The main message is that practice is consistent with Swedish guidelines which is of course evidence based. Although the study is well performed and the paper is well written, there are significant concerns.

 The paper does not address that transrectal biopsy is a severely contaminated procedure. All contaminated procedures should be replaced by clean procedures whenever possible. Prostate biopsy can now be performed via the transperineal route through disinfected skin in local anesthesia as an outpatient procedure.

 The paper does also not address the new regulations by EMA which states that fuoroquinolones should not be used for prophylaxis.

 Extended prophylaxis will reduce the rates of infective complications but violates the principles of antimicrobial stewardship.

 Finally, urology departments should have been asked about the local resistance rates to fluoroquinolones.

 Our clinical practice should always be based on established surgical principles of hygiene. If we do that we will also comply with the principles of antimicrobial stewardship. These principles outweigh evidence from studies comparing one antibiotic with another. Having said this, there is increasing evidence that transperineal targeted as well as systematic biopsies can be performed safely without antibiotic prophylaxis.

 Swedish guidelines as well as international guidelines on prostate biopsies need to be updated.

Is the work clearly and accurately presented and does it cite the current literature?

Yes

If applicable, is the statistical analysis and its interpretation appropriate?

Yes

Are all the source data underlying the results available to ensure full reproducibility?

Yes

Is the study design appropriate and is the work technically sound?

Yes

Are the conclusions drawn adequately supported by the results?

Yes

Are sufficient details of methods and analysis provided to allow replication by others?

Yes

Reviewer Expertise:

Hospital acquired infections in urology

I confirm that I have read this submission and believe that I have an appropriate level of expertise to confirm that it is of an acceptable scientific standard.
